# Complete genome sequence of *Microbacterium foliorum* phage Curie, a podovirus isolated from soil in Spokane, Washington

**DOI:** 10.1128/mra.00408-24

**Published:** 2024-07-22

**Authors:** Emma N. Horton, Erika K. Beach, Kathryn T. Cook, Kyra G. Cronin, Avery T. Haag, Sierra M. Salter, Nicole A. Stojanovic, Zoe E. Fry, Brian M. Connolly, Rebekah F. Hare, Ann-Scott H. Ettinger, Marianne K. Poxleitner, Kirk R. Anders

**Affiliations:** 1 Department of Biology, Gonzaga University, Spokane, Washington, USA; Loyola University Chicago, Chicago, Illinois, USA

**Keywords:** bacteriophages

## Abstract

Bacteriophage Curie is a podovirus that infects *Microbacterium foliorum*. The Curie genome spans 16,810 bp, has 90 bp terminal inverted repeats, and includes 23 protein-coding genes. Its genome architecture resembles phage PineapplePizza and other phi29-like phages. Together, Curie and PineapplePizza form a new actinobacteriophage Cluster GI.

## ANNOUNCEMENT

Studying the genomic diversity of bacteriophages provides insight into their biology and evolution (1 – 3), and may also contribute to development of therapies against antibiotic-resistant bacterial infections (4). To explore the diversity of phages that can infect actinobacteria, we isolated phage Curie on *Microbacterium foliorum*. Here, we describe its genome.

**Fig 1:**
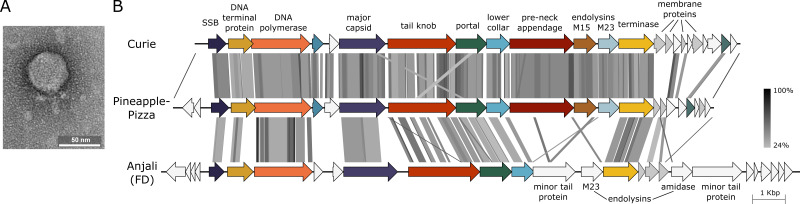
Phage Curie virion and genome structure. (**A**) Transmission electron micrograph of Curie, negatively stained with 1% uranyl acetate. (**B**) Curie genome compared to PineapplePizza and Anjali, a representative of Cluster FD. Gray bands between genomes show tBLASTx (v.2.15) alignments (7). The shade of each band indicates percent identity according to the grayscale bar, 24%–100%. Genes (arrows and arrowheads) are filled with the same color if they were members of gene families formed by Phamerator (8), using sequences in the Actino_Draft database version 552 (9). Genes filled with gray encode proteins with at least one predicted transmembrane domain. Endolysins are labeled with the domain they contain: M15 peptidase, M23 peptidase, or N-acetylmuramoyl-L-alanine amidase. The genome alignment was generated with EasyFig v.2.2, filtering for tBLASTx e-values smaller than 10^−50^ (10).

Fifteen cubic centimeters of top layer soil was collected in Spokane, WA (47.66669 N, 117.40284 W; September 2021), incubated 7 days in 35 mL PYCa medium (5) seeded with 0.5 mL of a saturated, 2-day batch culture of *Microbacterium foliorum* NRRL B-24224, then filtered (0.22 µm). Plaques were found on lawns grown from 0.5 mL saturated *M. foliorum* culture and 10 µL phage solution in PYCa top agar on a PYCa base and incubated 2–7 days at 22°C (6). An isolated plaque was picked and replated three times, and a lysate was collected (6). Transmission electron microscopy revealed a podoviral morphology with average capsid width of 46 nm and tail length of 27 nm (Fig. 1A, *n* = 3).

DNA was extracted from the lysate with the Promega DNA Wizard Clean-Up System. A library was made with the NEB Ultra II FS kit (v.3 reagents) and sequenced with Illumina MiSeq, producing 290,051 single-end, 150-base reads that were assembled with Newbler v.2.9 (11). Consed v.29 (12) was used to assess and finish the assembly, which had an average coverage depth of 2,141×, resulting in a genome of 16,810 bp with terminal inverted repeats of 90 bp. The G + C content was 50.1%, distinct from *M. foliorum* DNA at 68.7% (13). As no sequencing reads extended beyond the ends, a terminal protein may be covalently attached, similar to phi29 (14). Curie was most closely related to PineapplePizza (GenBank ON724010), sharing an average nucleotide identity of 63% (DNA Master v.5.23, http://cobamide2.bio.pitt.edu) (Fig. 1B). Curie joined PineapplePizza to form actinobacteriophage Cluster GI, based on nucleotide sequence similarity and shared gene content (8, 9, 15, 16).

Twenty-three protein-coding genes were predicted using GeneMark v.2.5p (17), Glimmer v.3.02 (18), Starterator v.1.2 (https://github.com/cdshaffer/starterator), and Phamerator (8). No tRNA genes were detected with Aragorn v.1.2 (19) or tRNAscan-SE v.2.0 (20). Gene functions were predicted using HHPred alignments in PDB, Pfam, SCOP, and CDD databases (21 – 25), NCBI BLAST v.2.14 (7), the Actinobacteriophage Database (9), and DeepTMHMM v.1.0 (https://dtu.biolib.com/DeepTMHMM). (All software used default parameters, unless noted.) Curie, PineapplePizza, and phages in Cluster FD such as Anjali (MK016490) share a similar core genome architecture (Fig. 1B). The shared region extends from the ssDNA-binding protein gene on the left to terminase on the right, although Anjali and its relatives (isolated on *Arthrobacter globiformis*) carry different minor tail and endolysin genes (Fig. 1B). The shared genes are similar to those of the podovirus phi29 (26), whose virion structure is well-described (27). Curie and the other phi29-like genomes contain 10–13 small genes near the genome ends, several of which may encode transmembrane domains (Fig. 1B). Although we detected no sequence similarity with phi29 holin, we speculate that one or more of the membrane proteins functions as a holin.

## Data Availability

The complete genome sequence of Curie has been deposited in DDBJ/ENA/GenBank under the GenBank accession number PP059645 and Sequence Read Archive accession number SRX23452930. The GenBank genome version described in this paper is the first version, PP059645.1.
